# Comparative Transcriptome Analysis to Investigate the Immunotoxicity Mechanism Triggered by Dimethomorph on Human Jurkat T Cell Lines

**DOI:** 10.3390/foods11233848

**Published:** 2022-11-28

**Authors:** Yun-Cheng Li, Shu-Yan Liu, Fan-Bing Meng, Shu-Hui Xu, Jing Qiu, Yong-Zhong Qian, Yan-Yang Xu, Yun Li

**Affiliations:** 1 Institute of Quality Standards and Testing Technology for Agro-Products, Chinese Academy of Agricultural Sciences, Beijing 100081, China; 2 College of Food and Biological Engineering, Chengdu University, Chengdu 610106, China

**Keywords:** dimethomorph, Jurkat T cells, immunotoxicity, transcriptomics, RNA sequencing

## Abstract

Dimethomorph (DMM) is a broad-spectrum fungicide used globally in agricultural production, but little is known regarding the immunotoxicity of DMM in humans. In this study, the immunotoxicity of DMM on human Jurkat T cells was evaluated in vitro. The results indicated that the half-effective concentration (EC_50_) of DMM for Jurkat cells was 126.01 mg/L (0.32 mM). To further elucidate the underlying mechanism, transcriptomics based on RNA sequencing for exposure doses of EC_25_ (M21) and EC_10_ (L4) was performed. The results indicated that compared to untreated samples (Ctr), 121 genes (81 upregulated, 40 downregulated) and 30 genes (17 upregulated, 13 downregulated) were significantly differentially regulated in the L4 and M21 samples, respectively. A gene ontology analysis indicated that the significantly differentially expressed genes (DEGs) were mostly enriched in the negative regulation of cell activities, and a KEGG pathway analysis indicated that the DEGs were mainly enriched in the immune regulation and signal transduction pathways. A quantitative real-time PCR for the selected genes showed that compared to the high-dose exposure (M21), the effect of the low-dose DMM exposure (L4) on gene expression was more significant. The results indicated that DMM has potential immunotoxicity for humans, and this toxicity cannot be ignored even at low concentrations.

## 1. Introduction

With the deterioration of the environment and the growth of the population, a good public health environment and adequate food supplies have increasingly become stumbling blocks for the development of human society [1,2,3]. Pesticides play an important role in controlling insect-borne diseases and developing agricultural production [2,4,5]. The ideal pesticide is effective against targeted diseases and insect pests without harming the human body; however, this seems difficult to achieve [6]. Mounting evidence has shown that pesticide residues can impact human health through environmental and food contamination [2], even at very low levels of exposure [4]. Therefore, a comprehensive understanding of pesticide toxicities is essential to the rational application of pesticides.

Numerous pesticide toxicity assessments have been carried out in recent years, but most of them are focused on general toxicity (acute toxicity, subchronic or subacute toxicity, chronic toxicity, etc.), endocrine toxicity, neurotoxicity, and “mutagenesis, carcinogenesis, teratogenesis” effects [7,8,9,10]. However, when a chemical compound is stated to be toxic, it does not necessarily mean that it induces the death of cells; effects may not result in cytotoxicity but alteration of cell function, leading to a detrimental outcome [11]. Among them, the immune system is the first line of defense against foreign hazardous chemicals within the human body. Thus, the immune response triggered by pesticides may be closely associated with the predisposition to different types of disease because the immune system mutually and closely interacts with all body organs [12,13]. Therefore, immunotoxicity evaluations of pesticide residues are very important for a comprehensive pesticide residue risk assessment that can guide agricultural production. Traditional immunotoxicity is usually evaluated through animal experiments for studying the specific antigen immune response, immune function, and so on. However, animal experiments are time consuming and require a lot of animal materials, and the use of animals is an important ethical and political issue [14]. Therefore, animal alternative methods (such as in vitro cell experiment) combined with bioinformatics has the potential to provide more comprehensive knowledge on the toxicological mechanism of chemicals in biological systems than more traditional approaches [15].

Dimethomorph (4-[3-(4-chlorophenyl)-3-(3-4-dimethox-yphenyl) acryloyl] morpholine, DMM), a cinnamic acid derivative, is a broad-spectrum fungicide globally used in agricultural production to prevent gray mold, powdery and downy mildews, crown and root rots, and late blight [16,17]. Since the use of DMM is very extensive, some reports in recent years have shown that the content of water, soil, and agricultural products of DMM range from ng/kg to mg/kg [18] and pose a certain risk to living organisms, including aquatic organisms, birds, and mammals [19,20]. Therefore, numerous studies have been performed to determine the dissipation and residue of DMM in vegetables, fruits, and their processed products [19,20,21,22]. More importantly, many studies have demonstrated that DMM is toxic to some soil and water microflora, birds, and mammals, even at very low concentrations [18,20]. Although the Environmental Protection Agency (EPA) reports show that DMM has low toxicity to humans, there has been insufficient knowledge regarding the toxicity and toxicity mechanisms of DMM pesticides in humans, especially immunotoxicity. In addition, owing to the continual and prolonged exposure of dimethomorphs, previous studies have indicated that fungal species have developed resistance and become insensitive to lower concentrations of DMM [16]. In order to fight fungal infection, a higher concentration of DMM is sprayed in fields, resulting in an increase in residues within agricultural products, as high as 7 mg/kg, which has been detected in vegetables. Meanwhile, a previous study also suggested that DMM is extremely resistant to hydrolysis and has a long half-life in the ecosystem [18]. Therefore, it is necessary to study the toxicity and mechanism of DMM in order to provide some reference for its risk assessment.

In the present study, the in vitro immunotoxicity of DMM in humans was investigated by using the human Jurkat T cell line, an in vitro model system frequently used in immunotoxicity evaluation due to its well-established reliability [23]. In addition, a comparative transcriptome analysis was applied to reveal the underlying immunotoxicity mechanism of DMM. To our knowledge, this is the first study to focus on the immunotoxicity of DMM to human immune cells, and the results provide a reference for the risk assessment of DMM.

## 2. Materials and Methods

### 2.1. Chemicals and Reagents

Dimethomorph (DMM, 99.9% purity) was obtained from A Chemtek Inc. (Worcester, MA, USA). The human T-lymphocyte cell line (Jurkat T cells) was obtained from the American Type Culture Collection (ATCC, Manassas, VI, USA); this cell line was derived from the peripheral blood of human T-lymphocyte leukemia cells. Acetone (HPLC grade) was purchased from Merck & Co. (Darmstadt, Germany). RPMI-1640 medium, penicillin/streptomycin, phosphate-buffered saline (PBS), and fetal bovine serum (FBS) were all purchased from HyClone (Logan, UT, USA). The Cell Counting Kit-8 was purchased from Dojindo (Kumamoto, Japan). The Annexin V-FITC/PI detection kit was purchased from Abbkine (Wuhan, China). The Mycoplasma Stain Kit was purchased from Sigma Aldrich (St Louis, Missouri, MO, USA), the TruSeq^TM^ RNA Sample Preparation Kit was purchased from Illumina (San Diego, CA, USA), and the PrimeScript RT Reagent Kit was purchased from Beyotime Biotechnology (Shanghai, China). Unless otherwise specified, the reagents used in this study were of analytical grade.

### 2.2. Jurkat Cell Culture

As DMM has low solubility in water, an 11,000 mg/L stock solution of dimethomorph was prepared in acetone without FBS and maintained at −20 °C [24]. Final concentrations of DMM in the assay were achieved by their dilution in the culture medium. The final acetone concentration in the medium was less than 0.1% (*v*/*v*). The Jurkat cells were inoculated in RPMI-1640 medium containing 10% (*v*/*v*) heat-inactivated FBS, 100 U/mL of penicillin sodium, and 100 μg/mL of streptomycin solution, and incubated in a humidified atmosphere containing 5% CO_2_ at 37 °C. The cells were kept at the logarithmic phase by passages at 2–3 d intervals. The absence of mycoplasma was routinely checked using the Mycoplasma Stain Kit [25].

### 2.3. Cell Viability Assay

Cell viability was assayed according to a previous study with some modifications [26]. Briefly, activated Jurkat T cells were diluted to 2 × 10^5^ cells/mL using fresh medium, pipetted into 100 μL of the cell dilutions, seeded in a 96-well multiplate, and treated for 36 h with DMM at final concentrations of 0.5, 5, 25, 50, 100, 250, and 500 mg/L. The final acetone concentration of each well was adjusted to the same concentration and less than 0.1%, which exerted no effect on cell viability. A blank group (without pesticide and cells) and a control group (containing cells, equivalent solvent but without pesticide) were included. Cell viability was determined by the Cell Counting Kit-8 (CCK-8), according to the manufacturer’s instructions. Absorbance was measured at 450 nm in a ReadMax 500F enzyme-labeled instrument (Shanpu Biotechnology Co., Ltd., Shanghai, China). Cell viability was calculated using Equation (1).

(1) $$\mathrm{Cell}\;\mathrm{viability}=\frac{A_{t}-A_{b}}{A_{c}-A_{b}}$$

where A_t_ is the absorbance of the test group, A_b_ is the absorbance of the blank group, and A_c_ is the absorbance of the control group. Concentration–response curves were plotted, and the half maximal effective concentration (EC_50_) values were then calculated using a sigmoidal dose–response curve equation [27].

### 2.4. Cell Apoptosis Analysis

Cell apoptosis was assessed by using an Annexin V-FITC/PI detection kit. Activated Jurkat T cells were diluted to 2×10^5^ cells/mL using fresh medium, pipetted into 4 mL of the cell dilutions, seeded in a 6-well multiplate, and treated for 36 h with DMM at final concentrations of EC_50_, EC_25_, EC_10_, and the control group. The cells were collected and washed to remove the medium, resuspended in binding buffer, and incubated with Annexin V-FITC solution and PI solution at normal temperature for 15 min. Apoptotic cells were analyzed by a MoFlo Astrios^EQ^ flow cytometer (Beckman Coulter, Inc., Brea, CA, USA) [28].

### 2.5. Transcriptome Analysis

#### 2.5.1. RNA Extraction and High-Throughput Sequencing

The Jurkat T cells were seeded in a 75 cm^2^ cell culture bottle with 60 mL of medium at an initial concentration of 1.2 × 10^7^ cells/bottle and treated for 36 h with DMM at final concentrations of 4 mg/L (EC_10_) and 21 mg/L (EC_25_). The cells were collected for transcriptome analysis. Total RNA was isolated using the TRIzol^®^ reagent (Thermo Fisher, Waltham, MA, USA), according to the manufacturer’s protocol, and genomic DNA was removed using DNase I (TaKaRa, Dalian, China). Then, RNA quality was determined by a 2100 Bioanalyzer (Agilent Technologies, Santa Clara, CA, USA) and quantified using an ND-2000 (NanoDrop, Wilmington, DE, USA). Only high-quality RNA samples (OD260/280 = 1.8~2.2, OD260/230 ≥ 2.0, RIN ≥ 6.5, 28S:18S ≥ 1.0, >1 μg) were used to construct a sequencing library.

The RNA transcriptome library was prepared following the TruSeq^TM^ RNA sample preparation kit. Libraries were size selected for cDNA target fragments of 300 bp on 2% Low Range Ultra Agarose, followed by PCR amplification using Phusion DNA polymerase (NEB, Ipswich, MA, USA) for 15 PCR cycles. After quantification by TBS380, a paired-end RNA sequencing library was obtained by using a Nova Seq 6000 sequencer (2 × 150 bp read length).

#### 2.5.2. Read Mapping and Differential Expression Analysis

The raw paired-end reads were clipped and quality controlled by SeqPrep https://github.com/jstjohn/SeqPrep (accessed on 10 January 2022) and Sickle https://github.com/najoshi/sickle (accessed on 10 January 2022) with the default parameters. The clean reads of each sample were sequenced and aligned with the specified reference genome Homo_sapien, http://asia.ensembl.org/Homo_sapiens/Info/Index (accessed on 23 January 2022). The mapped reads of each sample were assembled by StringTie https://ccb.jhu.edu/software/stringtie/index.shtml t = example (accessed on 23 January 2022) in a reference-based approach [29].

To identify the differential expression genes (DEGs) between the two different samples, the expression level of each transcript was calculated according to the fragments per kilobase of exon per million mapped fragments (FPKM) method. RSEM http://deweylab.biostat.wisc.edu/rsem/ (accessed on 20 March 2020) [30] was used to quantify gene abundances. A differential expression analysis was performed using DESeq2 [31] with |log_2_FC| > 1.3, and a *Q*_value_ ≤ 0.05 was considered to indicate significantly differentially expressed genes. Functional enrichment analyses, including gene ontology (GO) and Kyoto Encyclopedia of Genes and Genomes (KEGG) analyses, were implemented to find significantly enriched DEGs in GO terms and metabolic pathways at a Bonferroni-corrected *P*_value_ ≤0.05 compared with the whole-transcriptome background [32].

### 2.6. Quantitative Real-Time PCR

Four genes that were significantly differentially expressed were selected for QRT-PCR analysis, and GAPDH was used as the reference gene. The primers were designed with Primer-BLAST http://www.ncbi.nlm.nih.gov/tools/primer-blast/ (accessed on 3 November 2020) and are presented in Supplementary Table S1. Total RNA was reverse-transcribed using the PrimeScript RT Reagent Kit with gDNA Eraser. The reactions were prepared on a StepOne Plus^TM^ Real-time PCR detection system (ABI, Boston, MA, USA) with a total volume of 10 μL: 3 μL of 1:2 diluted template, 1 μL of each primer (5 μM), and 5 μL of 2× Fast SYBR^®^ Green Master Mix (ABI, Boston, MA, USA). Baseline, threshold cycles (Ct), and statistical analyses were automatically determined using the StepOne Plus^TM^ Software version 2.3 (ABI, Boston, MA, USA).

### 2.7. Statistical Analysis

The cell viability assay was tested in three independent experiments with five biological replicates; the cell apoptosis analysis was tested in three independent experiments; the transcriptome analysis was tested in three independent experiments with three biological replicates. Data are expressed as the mean ± SD of three independent experiments. All statistical analyses were performed using SPSS version 18.0 software (IBM). The values were compared with a one-way ANOVA followed by Duncan’s test. *p* < 0.05 was considered statistically significant.

## 3. Results and Discussion

### 3.1. Effect of Dimethomorph on Cell Viability

**Figure 1:**
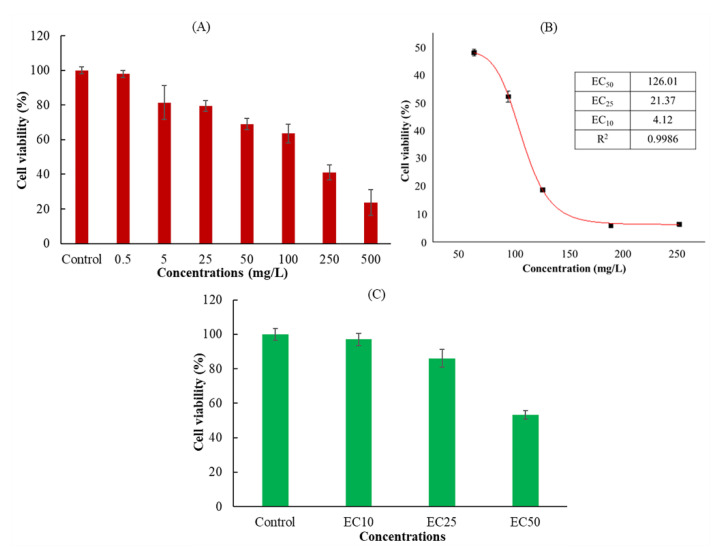
Effect of dimethomorph on Jurkat T cells viability. (**A**) Jurkat cells exposed for 36 h at different DMM concentrations or controls. (**B**) Nonlinear curve fitting results of different effective concentration (EC) using the results of (**A**). (**C**) Jurkat T cells exposed for 36 h to EC_50_, EC_25_, EC_10_ DMM, or controls. Cell viability is presented as a percentage compared to the control. The results shown are the mean ± SD from triplicate exposures.

The action mechanism of DMM is to destroy the cell wall membrane, causing the decomposition of the sporangium wall and inducing pathogen death [33]. However, the immunotoxicity of DMM on the human body and its mechanism has not received much attention. In the process of in vitro cytotoxicity evaluation, cell proliferation is an important marker for the evaluation of cytotoxicity [34]. The assessment of cellular activity was based on the ability of these cells to metabolize water-soluble tetrazole-8 (WST-8) of CCK-8 and convert it to orange formazan via mitochondrial dehydrogenase. Cell viability was determined by the extent of WST-8 cleavage by mitochondrial dehydrogenases in DMM-exposed cells and controls [35]. Figure 1A shows the viability of Jurkat cells after exposure to different concentrations of DMM. Compared to the control group (with 0.1% acetone but without DMM addition), the cell activity decreased with an increasing DMM concentration, showing a concentration-dependent trend. In the EC_50_, EC_25_, and EC_10_ treatment groups, the Jurkat cells calculated by nonlinear curve fitting were 126.01 mg/L (0.32 mM), 21.37 mg/L (0.06 mM), and 4.12 mg/L (0.01 mM), respectively (Figure 1B). When the cells were exposed to the EC_50_, EC_25_, and EC_10_ treatment groups, the cell activities were consistent with the expected results (97.8%, 81.8%, and 53.4%, respectively) (Figure 1C), which could be used in subsequent apoptosis experiments.

### 3.2. Effect of DMM on Cell Apoptosis

**Figure 2:**
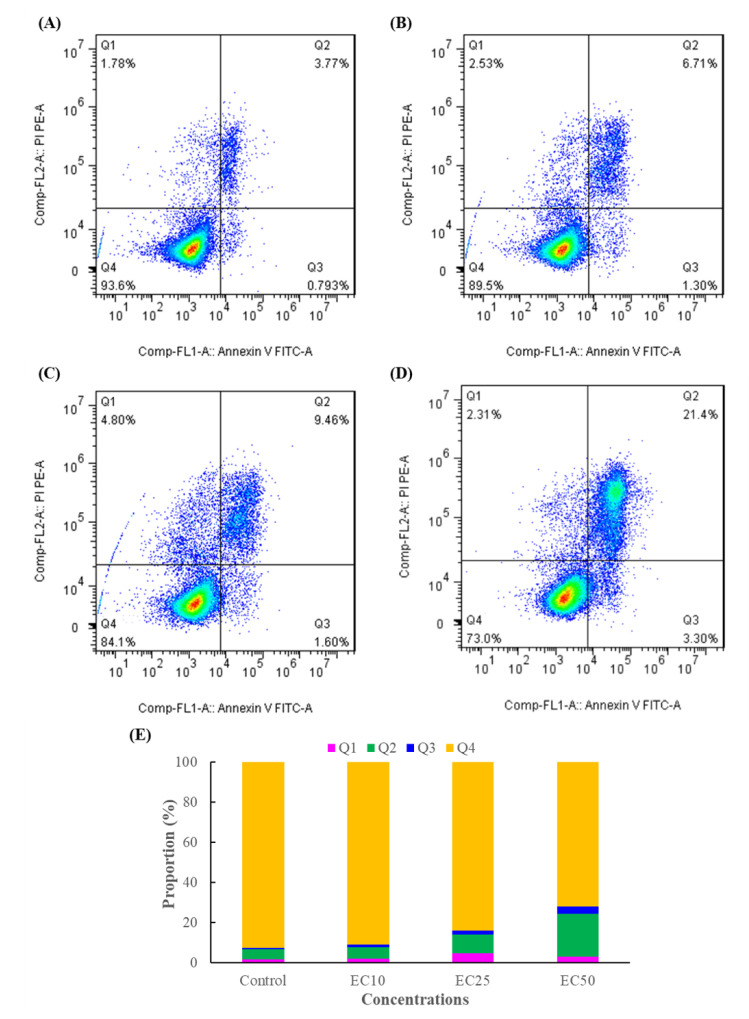
DMM-induced apoptosis in Jurkat T cells. (**A**): Control, (**B**): EC_10_, (**C**): EC_25_, (**D**): EC_50_, (**E**): statistics of the apoptosis data of three independent experiments. Q1, Q2, Q3, and Q4 of the flow cytometry graph indicate dead cells, late apoptotic cells, early apoptotic cells, and normal cells, respectively.

Previous studies have indicated that cell apoptosis or programmed cell death is closely linked to cell proliferation in mammalian cells [36], and the Annexin-V/PI staining assay is a simple and effective method to detect apoptosis at a very early stage [3]. From the results of Figure 2A–D, there was no significant change in apoptosis between EC_10_ and the control group, but with the increasing DMM concentration, the ratio of the late apoptotic cells for the EC_25_ and EC_50_ treatments increased 1.93-, 4.37-fold higher than that of the control. The results indicated that DMM caused the apoptosis of Jurkat T cells in a concentration-dependent manner, which is consistent with the results predicted in the cell viability experiment, and could be used in subsequent experiments.

### 3.3. RNA Extraction and Quality Evaluation

**Figure 3:**
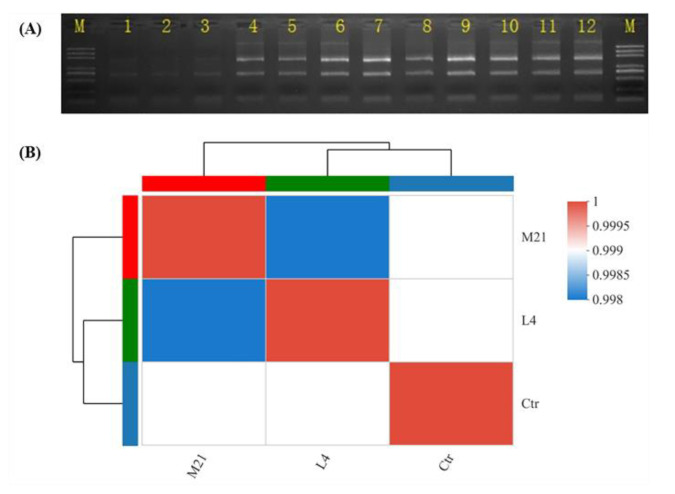
RNA electropherogram (**A**) and sample correlations based on RNA sequencing (**B**). (**A**): M, marker; 1–3, EC_50_ group; 4–6, EC_25_ group; 7–9, EC_10_ group; 10–12, control group.

In the in vitro immunotoxicity screening test using Jurkat T cells, DMM was found to have significant immunotoxicity. Therefore, we further systematically evaluated the immunotoxicity of DMM on human Jurkat T cells, and the mechanism of action was also expounded by using comparative transcriptomics. To fully elucidate the underlying mechanism, a transcriptome analysis based on RNA-seq was performed. During transcriptomic studies, selecting a proper pesticide exposure concentration is very important, because too high a concentration could cause cell death, and too low a concentration might not be cytotoxic [37]. As shown in Figure 3A and Table 1, when the exposure concentration was EC_50_, the RNA bonds were unclear, and the RIN was below 8, which indicated that the total RNA degraded to a degree that it was not suitable for transcriptome analysis [38]. The RNA bonds under the EC_25_ treatment (named M21), the EC_10_ treatment (named L4), and the control (named Ctr) were clear, and there was no contamination of other impurities. The RIN values were higher than 9.5, which indicates good RNA quality. Moreover, according to the procedure of the ISO 10993-5 standard, a tested material that is incubated for at least 24 h with precultured cells and has a decreased viability of under 70% of the control is considered cytotoxic [39]. As shown in Figure 1, the cell viability under the EC_10_ and EC_25_ treatments exposure was 97.8% and 81.8%, respectively, which indicated that samples M21 and L4 could be used in the following transcriptomic analysis.

### 3.4. RNA Sequencing Data Assessment

In this study, a total of 77.76 Gb of high-quality clean reads were obtained after the unqualified reads were filtered out. The clean reads of each sample in each group reached more than 7.63 Gb, and the sequencing error rate was less than 0.025%. The Q30 base accounted for more than 94.18% and the GC content ranged from 49.4% to 50.36% (Table 2). The statistics indicate that the quality of the sequencing is high enough for further analysis. As shown in Figure 3B, the correlation coefficients between samples (Ctr group, L4 group, and M21 group) were higher than 99%, and the phylogenetic tree analysis results demonstrated that the Ctr group was different from the L4 group and the M21 group, but there was a high correlation between the control group and the L4 group. These results are consistent with expectations, so the results revealed good reliability among the samples [40].

### 3.5. Gene Expression Overview

**Figure 4:**
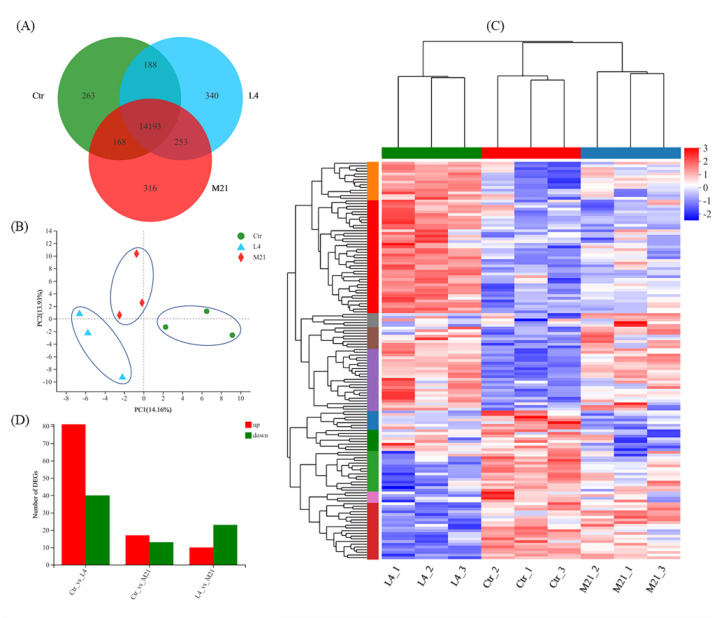
Venn diagram (**A**) and principal component analysis (PCA) for the differentially expressed genes (**B**). Hierarchical clustering analysis (HCA) of significantly differentially expressed genes (**C**) and statistics of the significantly differentially expressed genes (**D**) for each comparison.

As shown in Figure 4A, a total of 15,721 genes were identified through RNA sequencing, and 14,193 genes were co-expressed in the Ctr, L4, and M21 samples. A principal component analysis (PCA) was performed to assess the transcriptomics of the different samples. Figure 4B reveals that the Ctr, L4, and M21 samples were well divided into three characteristic groups by PCA, which indicated that there were significant differences in the transcriptomics between the groups [41]. Further analysis indicated that compared to the Ctr samples, 121 genes (81 upregulated, 40 downregulated) and 30 genes (17 upregulated, 13 downregulated) were significantly differentially regulated in the L4 and M21 samples, respectively (Figure 4C,D, Tables S2 and S3). The results indicated that DMM can significantly interfere with the gene expression of Jurkat T cells, even at a low dose (4.12 mg/L L4 group). This result is consistent with that of the toxicity test shown in Figure 1. Previous studies showed that the highest dimethomorph residue was 6.8 mg/kg for leafy vegetables and stalk and stem vegetables and 6.11 mg/kg for *Dendrobium officinale* [42,43]. Considering the cytotoxicity and transcriptomic results of our study, the immunotoxicological effects of DMM should be emphasized.

### 3.6. Gene Ontology (GO) Analysis of Differentially Expressed Genes (DEGs)

**Figure 5:**
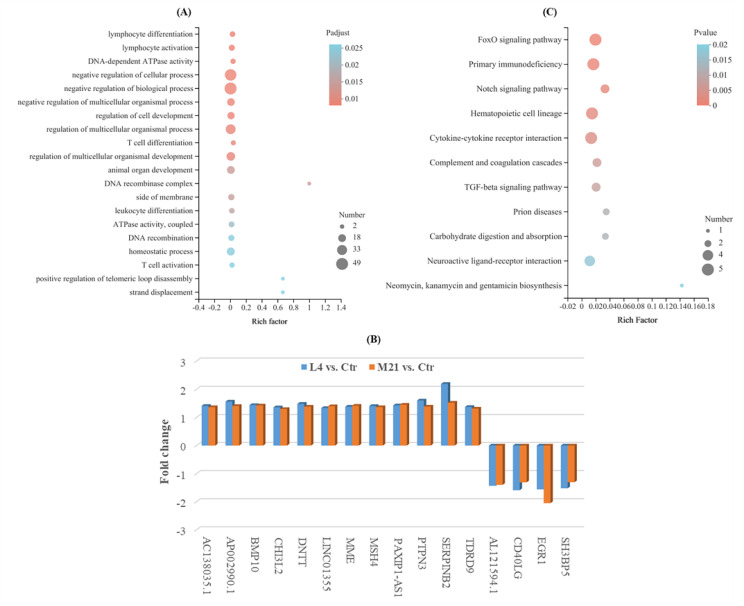
Gene ontology enrichment analysis for the significantly differentially expressed genes (**A**), simultaneously significantly different expressed genes for both comparison of L4 vs. Ctr and M21 vs. Ctr (**B**), and KEGG enrichment analysis for the significantly differentially expressed genes (**C**).

A GO enrichment analysis can be used to reveal the functional characteristics of differentially expressed genes (DEGs). GO terms are widely used to classify genes into the categories of cellular component (CC), molecular function (MF), and biological process (BP) [44]. As shown in Figure 5A, the GO enrichment analysis indicated that the DEGs were mostly enriched in biological processes (involving 31 BPs, *P*_adjust_ ≤0.05) (Supplementary Table S4), and many of them were involved in the negative regulation of cell activities, such as the negative regulation of biological processes, cellular processes, and cell development, which indicated that DMM has negative toxic effects on Jurkat T cells. Moreover, many genes were also involved in the immune regulation of cell biological processes, such as lymphocyte activation and differentiation, T cell activation and differentiation, leukocyte differentiation, and negative regulation of the T cell apoptotic process. Most of the genes were downregulated in comparison to L4 vs. Ctr or M21 vs. Ctr (such as *RAG1*, *HDAC9*, *SOX4*, and *CD7*). However, only 12 genes (*AC138035.1*, *AP002990.1*, *BMP10*, *CHI3L2*, *DNTT*, *LINC01355*, *MME*, *MSH4*, *PAXIP1-AS1*, *PTPN3*, *SERPINB2*, and *TDRD9*) were upregulated, and four genes (*AL121594.1*, *CD40LG*, *EGR1*, and *SH3BP5*) were downregulated simultaneously in comparison to both L4 vs. Ctr and M21 vs. Ctr (Figure 5B). Among them, two important genes, *EGR1* and *CD40LG*, related to immune regulation were downregulated in both comparisons to L4 vs. Ctr and M21 vs. Ctr. Human CD40LG protein is a transmembrane protein and the ligand of CD40. It belongs to the tumor necrosis factor gene superfamily and is involved in immune-related pathways of breast cancer. The gene *CD40LG* plays critical roles in the regulation of the activation and differentiation of B cells and the maturation of dendritic cells [45]. The gene *EGR1* (early growth response 1) is an important transcription factor that is widely expressed in many cell types and participates in important physiological processes of human cells [46]. A previous study indicated that *EGR1* serves as a tumor suppressor in cancers, such as prostate tumors and gastric tumors [47]. The downregulation of these genes may indicate that DMM exposure reduces the immune resistance of the body, even at a low concentration.

### 3.7. KEGG Enrichment Analysis of DEGs

KEGG is a database for the systematic analysis of gene function and genome information, which can be used as a whole network to study gene and expression information [48]. As shown in Figure 5C and Supplementary Table S5, the significant DEGs were mainly enriched in 11 KEGG pathways (*P*_value_ ≤ 0.05), and most of them were related to immune regulation and signal transduction. Among them, the hematopoietic cell lineage, hematopoietic cell lineage, the FoxO signaling pathway, and the cytokine-cytokine receptor interaction are closely related to the occurrence of cancer in humans. Cytokines are crucial intercellular regulators and mobilizers of cells engaged in innate and adaptive inflammatory host defenses, cell growth, differentiation, cell death, angiogenesis, and development and repair processes aimed at the restoration of homeostasis [49]. FOXO (Forkhead Box O) is a subgroup of Fox transcription factors that are considered to play a key role as tumor suppressors in a variety of cancers [50]. Complement and coagulation cascades could interact with systemic lupus erythematosus (SLE), and this interaction may lead to aggravation of the disease, which is more obvious in inflamed patients [51]. In the complement and coagulation cascades pathway, the expression of the important gene *SERPINB2* was increased in both L4 vs. Ctr and M21 vs. Ctr. A previous study showed that the protein SerpinB2 is substantially upregulated under multiple inflammatory conditions, and dysregulated expression and polymorphisms are associated with several human inflammatory diseases [52]. The above results further indicated that DMM exposure reduces the immune resistance of the body, even at a low concentration. DMM has certain immunotoxicity to the human body. Even low-dose exposure can cause immune reactions and cause potential harm to the body.

### 3.8. Target Gene Screening and Quantitative Real-Time PCR Validation

**Figure 6:**
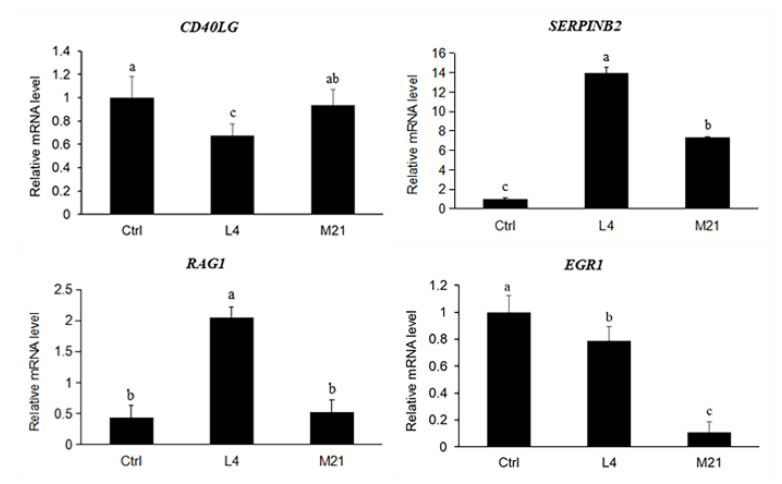
Quantitative real-time PCR results for the selected genes. Data are expressed as the mean ± SD from triplicate exposures. Different letters on the different bars indicate significant differences (*p* < 0.05).

To validate the results obtained from the transcriptome analysis, four important genes related to immune regulation were selected. As shown in Figure 6, there was general accordance between the RNA sequence and the real-time qPCR data for all the tested genes (*CD40LG* and *EGR1* downregulated, *SERPINB2* and *RAG1* upregulated), although the fold changes differed between the analytical methods. Compared to the high concentration exposure (M21), the effect of the low concentration DMM exposure (L4) on gene expression was more significant. Therefore, the low-dose chronic toxicity of DMM needs to be further studied.

## 4. Conclusions

This study demonstrated that when exposed to DMM, human Jurkat T cells’ activity decreased with increasing DMM concentration, and the half-effective concentration (EC_50_) of DMM for Jurkat cells was 126.01 mg/L (0.32 mM). There was no significant change in apoptosis between EC_10_ and the control samples, but the ratio of the late apoptotic cells for the EC_25_ and EC_50_ treatments increased 1.93-, 4.37-fold higher than that of the control. Transcriptomics based on RNA sequencing indicated that compared to untreated samples (Ctr), 121 genes (81 upregulated, 40 downregulated) and 30 genes (17 upregulated, 13 downregulated) were significantly regulated when exposed to EC_10_ (L4) and EC_25_ (M21), respectively. GO and KEGG analyses indicated that the DEGs were mostly involved in immune regulation and signal transduction pathways. The quantitative RT-PCR for the selected genes showed that the effect of low-dose DMM on gene expression was more significant than that of high-dose exposure. The results suggested that DMM exposure may cause immune system disturbance, and thus, negatively affect body health. In future, the in vivo experiments are necessary to further verify the immunotoxicity and target genes triggered by DMM.

## Figures and Tables

**Table 1:** RNA quality assessment.

Samples	Concentration(ng/μL)	Content (μg)	OD260/280	OD260/230	RIN
H126-1	115.00	4.03	2.05	2.11	7.40
H126-2	112.00	3.92	2.03	2.01	7.80
H126-3	96.20	3.37	2.04	2.10	7.60
M21-1	1545.90	54.11	2.02	2.21	10.00
M21-2	1731.70	60.61	2.01	2.18	9.90
M21-3	1678.50	58.75	2.01	2.17	10.00
L4-1	2141.30	74.95	1.99	2.14	10.00
L4-2	2090.50	73.17	1.99	2.13	10.00
L4-3	2133.00	74.66	2.00	2.13	9.50
Ctr-1	2020.60	70.72	2.00	2.15	9.90
Ctr-2	1862.20	65.18	2.01	2.17	9.60
Ctr-3	1892.30	66.23	2.01	2.18	9.90

RIN: RNA Integrity Number.

**Table 2:** Statistics and quality estimation of RNA-seq reads.

Sample	Raw Reads	Clean Reads	Clean Bases	Error Rate (%)	Q20 (%)	Q30 (%)
Ctr_1	69321808	68648290	10248272849	0.0247	98.15	94.41
Ctr_2	56231964	55657530	8295729777	0.0249	98.07	94.21
Ctr_3	62512340	61926524	9209389908	0.0245	98.25	94.67
L4_1	65369620	64580756	9635172686	0.0244	98.26	94.71
L4_2	51616818	51120046	7630992231	0.0246	98.21	94.57
L4_3	58748206	58167602	8680297728	0.0249	98.08	94.18
M21_1	52328192	51754638	7726615557	0.0249	98.07	94.23
M21_2	56519460	56045588	8350478112	0.0241	98.4	95.03
M21_3	54012066	53454568	7983424171	0.0248	98.1	94.31

## Data Availability

The data presented in this article are available on reasonable request, from the corresponding author.

## Supplementary Materials

The following supporting information can be downloaded at: https://www.mdpi.com/article/10.3390/foods11233848/s1, Table S1: Primer sequences of quantitative real-time PCR for the selected genes; Table S2: List of significantly differential expressed genes (DEGs) between L4 and Ctr; Table S3: List of significantly differential expressed genes (DEGs) between M21 and Ctr; Table S4: Gene ontology (GO) enrichment analysis of the DEGs; Table S5: KEGG enrichment analysis of the DEGs.
